# Distributed Acoustic Sensing for Future Planetary Applications: Initial Results From the San Francisco Volcanic Field, a Lunar Analogue

**DOI:** 10.1029/2024EA003640

**Published:** 2024-11-27

**Authors:** Nicholas Harmon, Ryan Porter, Catherine Rychert, Nicholas Schmerr, Madison M. Smith, Zhichao Shen, Wenbo Wu, Jacob Giles, Naoma McCall, Jingchuan Wang, Linden Wike, John West, Austin Hoyle, Naya Deykes

**Affiliations:** ^1^ Woods Hole Oceanographic Institution Woods Hole MA USA; ^2^ Northern Arizona University Flagstaff AZ USA; ^3^ University of Maryland College Park MD USA; ^4^ NASA Goddard Space Flight Center Greenbelt MD USA; ^5^ Arizona State University Tempe AZ USA

**Keywords:** distributed acoustic sensing, lunar seismology, refraction, MASW

## Abstract

Seismic imaging is one of the most powerful tools available for constraining the internal structure and composition of planetary bodies as well as enabling our understanding planetary evolution, geology, and distribution of natural resources. However, traditional seismic instrumentation can be heavy and voluminous, expensive, and/or difficult to rapidly deploy in large numbers. Distributed acoustic sensing (DAS) provides a promising new alternative given the ease of deployment, light weight and simplicity of fiber optic cables. However, the feasibility and best operational practices for using DAS for planetary exploration are not well‐known. We examine the use of DAS with surface deployed fiber for planetary near‐surface seismic exploration at two lunar geophysical analogue sites in San Francisco Volcanic Field. We compare DAS recordings to 3‐component seismometer recordings and geophone shot recordings and determine empirical response functions for the DAS system with respect to the 3‐component recordings. Shot sections of DAS and traditional seismic equipment compare well visually, with similar moveout of identifiable phases. DAS records first arrivals in good agreement with seismometers making them suitable for refraction work. Multichannel analysis of surface waves is performed on DAS records to estimate shallow shear velocities. The DAS has high spectral coherence with the horizontal components of ∼0.7 in the frequency band of the seismic shot energy. The empirical response functions are stable with amplitudes of ∼1.0–3.0 × 10^−10^ m per strain. Finally, the phase response is linear but not flat or zero. Our experiment demonstrates that there is potential for surface deployed DAS in planetary landscapes.

## Key Points

- Distributed acoustic sensing (DAS) with surface deployed fiber is tested for near surface active‐source seismic imaging for planetary studies
- Lunar analogue substrates require different fiber coupling strategies and highlight the need for optimizing deployment and DAS parameters
- DAS records compare reasonably with traditional seismic equipment, highlighting the potential for DAS in future planetary and lunar work

## 1 Introduction

Near surface geophysics provides valuable constraints to complement geologic field work on lunar and planetary bodies (Bell et al., 2022; Lognonné et al., 2020; NASA, 1971, 1972, 1973; Nunn et al., 2020). NASA's exploration of the Moon and Mars is being used to identify the potential for in situ resource utilization (ISRU) on these bodies, and geophysics is an important tool for determining the presence and abundance of near‐surface resources. For future missions, many of the resources capable of supporting ISRU for astronauts are localized in the near surface geology. A NASA Solar System Exploration Research Virtual Institute (SSERVI) called the Geophysical Exploration of the Dynamics and Evolution of the Solar System (GEODES) has a primary goal of understanding how geophysical tools and methods can be used to identify near‐surface resources (Schmerr et al., 2019). GEODES is investigating the geophysical detection and characterization of regolith, ice and volatiles, ore bodies, as well as void spaces and lava tubes, and studying the operational and instrumental requirements needed to conduct geophysical surveys in future crewed and robotic missions to the Moon and Mars. In addition to benefits for understanding surface geology, well constrained shallow structure can be used to enhance passive observations and disambiguate signals from shallow versus deep structure.

Prior successes demonstrate the utility of seismic imaging on planetary bodies using a small number of instruments. Active seismic experiments from the Apollo lunar missions constrained the compressional velocity structure and thickness of the shallowest regolith and thickness of geologic strata immediately beneath the surface layer for correlation with geologic observations (NASA, 1971, 1972, 1973; Nunn et al., 2020). The lunar active and passive data sets were also analyzed for shear velocity structure using H/V (horizontal/vertical) spectral ratios and Rayleigh wave analysis (Dal Moro, 2015; Mark & Sutton, 1975). The recent Interior Exploration using Seismic Investigations, Geodesy and Heat Transport (InSight) mission to Mars also successfully measured near‐surface structures using a variety of seismic techniques, including atmospheric compliance, receiver functions, and active source measurements (Banerdt et al., 2020; Carrasco et al., 2022; Lognonné et al., 2020). However, these efforts highlight the need for further studies incorporating arrays of instruments to achieve enhanced resolution and understanding (Bell et al., 2022; Léveillé, 2010; Panning et al., 2020).

Advances in the use of fiber optic cables using distributed acoustic sensing (DAS) provide a powerful new tool to supplement traditional seismic equipment (Lindsey & Martin, 2021; Zhan, 2019). DAS uses interferometry of back scattered laser light to measure strain, ε, along subsections of a fiber optic cable, effectively creating hundreds to thousands of virtual sensors along the fiber (Hartog, 2017; Lindsey & Martin, 2021). Due to the high number of sensors and spatial resolution DAS has been employed for use in a variety of engineering, geotechnical, and broader scale seismic applications. Distributed acoustic sensing investigations have frequently been performed on fiber embedded in oil and gas wells for m scale application (Daley et al., 2013, 2016) or using fiber in (“dark”) telecommunications cables on land (Ajo‐Franklin et al., 2019; Dou et al., 2017) or on the seafloor (Lindsey et al., 2019) for m‐km scale applications. However, fiber optic cables have also been deployed at the surface or in shallow trenches and shown to be effective for both active source (cm‐m scale applications) (Harmon et al., 2022; Spikes et al., 2019) and passive source seismic applications (> m scale) (Klaasen et al., 2023; Mjehovich et al., 2023; Yang et al., 2022). While, the previously described planetary seismology efforts employed a small number of seismometers or geophones (1–4), DAS offers potentially thousands of broadband sensor elements over km scales. The ability to rapidly deploy fiber optic cables and to record the seismic wavefield at high spatial and temporal resolution also make it an attractive technology for lunar and planetary studies (Wu et al., 2024).

Many of the remaining unknowns and challenges for surface/near‐surface deployment of DAS systems can be addressed through terrestrial analog experiments. The instrument response of DAS to ground motion and whether DAS can record wavefields with differing polarities with high fidelity, for example, P‐waves, S‐waves, Rayleigh, Love waves, etc., remain areas of active research (Jousset et al., 2018; Kuvshinov, 2016; Li et al., 2023; Lindsey et al., 2020; Reinsch et al., 2017). Open questions also remain around optimal coupling techniques; weighting, pinning and shallow burial have been used, with pros and cons for each method in terms of time, labor and effectiveness (Harmon et al., 2022; Mjehovich et al., 2023; Spikes et al., 2019). Another factor is determining the optimal fiber optic cable to use. Fiber optic cable can be made very durable via steel jacketing or Kevlar, and can be tight buffered, where the fiber directly in contact with the jacket, or loose buffered, where the fiber can move within the jacket. Commonly with loose buffered fiber either there is filling within the jacket such as gel or no additional jacket filling. These materials and buffering likely affect the response of the fiber to ground motion (Dou et al., 2017; Lagakos et al., 1982; Spikes et al., 2019), and have trade‐offs in terms of durability, sensitivity, ease of deployment, cost, and weight. Overall, while a handful of studies have been performed to investigate these questions, these factors all likely vary depending on the study site and/or target of interest, and therefore more experiments are needed for a full understanding, particularly under conditions that simulate planetary bodies.

In this study, we present findings from experiments with surface deployed DAS in a region that has long been used as a lunar planetary analogue for training astronauts prior to the Apollo missions (Léveillé, 2010), the San Francisco Volcanic Field in northern Arizona, USA (Wolfe et al., 1987). The region consists primarily of Pliocene to Pleistocene age basaltic material, with several major volcanic centers, each with lava flows and vent deposits (Wolfe et al., 1987). Within the region, two different field sites provide opportunities for evaluating techniques on varying substrates from lava flows to cinder/ash deposits. One of the sites is Lava River Cave lava tube, and the other is Sunset Crater. At both sites, the fiber was deployed concurrently with vertical geophones and 3‐component broadband seismometers. A range of coupling methods were used to adapt to the different substrates. Active source experiments were performed at both sites, and we compare signals recorded by the DAS system to those recorded by the geophones and broadband seismometers. Overall, DAS systems show promise for refraction analysis and surface wave analysis alongside traditional seismic systems. The results give insight into field techniques for surface deployment of DAS, and we discuss directions for further study.

## 2 Methods

### 2.1 Experimental Setup

**Figure 1:**
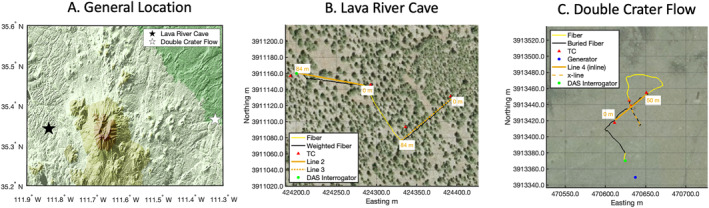
Map of the general location (a) Lava River Cave (b) and Double Crater Flow (c) with background SRTM topography (a) and aerial orthographic photography (b, c) (USGS, 2023). Panels (b, c) are in UTM coordinates in UTM zone 12. Legend indicates locations of fiber, TC, geophone lines 2,3,4, interrogators, and generator. There are some small differences in the fiber location (1–2 m) and the geophone lines that are likely due to small errors in the GPS locations. The fiber was typically within 10 cm of the geophones (See Figure 2).

**Figure 2:**
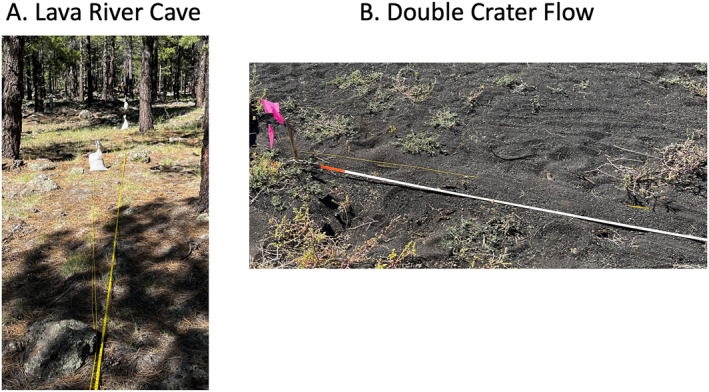
Coupling methods and substrate images. (a) Fiber at Lava River Cave was weighted under tension using 20 kg sandbags at ∼10 m intervals and supplemented with local rocks. The region is forested, with a surface covered by pine needles/light vegetation and underlying lava flow outcrops (b) The fiber at Double Crater Flow was shallowly buried (1–2 cm) in loose cinder and ash. Vegetation at the site is sparse, and the underlying lava flow was 10–20 cm deep below the cinder (Photos courtesy of Madison Smith).

Our experiments were performed at two different field sites. The Lava River Cave site was a pine forested area with pine needles over soil, and exposed boulders/regolith (Figures 1a and 2a). Beneath the site was a ∼1.2 km long lava tube which was 5–10 m in diameter for most of the cave. The lava tube is exposed at the surface near its entrance with the depth to the ceiling being of up to several meters beneath the surface. The second site was located on Double Crater Flow, which was a Pleistocene‐age flow with a thin covering of cinders from the 1085 CE Sunset Crater eruption (Alfano et al., 2018) (Figures 1b, and 2b). The substrate of this study area was loose cinder with small amounts of vegetation overlying a basalt flow. Each of these substrates provided uniquely challenging environments for surface deployments of fiber optic cable and traditional seismic equipment. Our experimental geometries were chosen to align with the planned work of the GEODES program at the worksites using a comprehensive suite of methods from electrical resistivity, gravity, ground penetrating radar, and active source seismology.

At Lava River Cave, there were two experiment lines that were used for active seismic and electrical resistivity: an 80 m roughly E‐W line (Line 2) as well as a 100 m NE‐SW trending line (Line 3) (Figure 1a). The lines were chosen by the GEODES program to cross and image the lava tube located several meters below the surface. A 290 m fiber optic cable was deployed to cover both lines with an ∼80 m section of cable connecting the two. We used 2 mm diameter, tight buffered single mode fiber with a plenum jacket because it was cost effective ($0.32/m), reasonably durable, and easy to transport. The fiber optic cable was laid out on the ground, stretched taut and weighted to the ground with ∼20 kg sandbags every 10 m along the two lines. The fiber was not weighted to the ground in the connecting region between the two lines (Figure 2a). In between sandbags, it was necessary to use local lava rocks as additional weights to ensure contact of the fiber with the ground, for example, where the fiber was suspended in the air. Geophones were simultaneously deployed along parts of the E‐W line and NE‐SW during the DAS recording period. Forty‐eight geophones were deployed at 0.5 m spacing, from 60.0 to 83.5 m on the E‐W line and from 0.0 to 23.5 m on the NE‐SW line. Four Nanometrics Trillium 120 Compact (TC) broadband seismometers were deployed using shallow burial (∼40 cm depth) along the lines as close as possible given terrain constraints (Figure 1b).

At the Double Crater Flow site, the GEODES experiment line was 50 m in length, oriented NE‐SW (Line 4) (Figures 1b, and 2b). The line was designed to image the thickness and layering of the lava flow. Two 290 m fiber optic cables were deployed from the interrogators, parallel along the line, and then looped back to create an additional perpendicular crossing line. The loose cinder and ash from nearby Sunset Crater was amenable to shallow burial of the fiber, so the fiber was covered with 1–2 cm of cinder after being stretched taut on the ground. The fiber was buried from the interrogators into the main line and cross line (x‐line), but the loop connecting the main line and cross line was unburied (Figure 2b). The fibers were in contact with each other at the crossing point, but physical contact did not seem to adversely affect the signals. Twenty‐four geophones were deployed along the main line for the duration of the experiment at a 2 m spacing (0–46 m), as were 4 TCs (Figure 1).

Two DAS interrogators were used: a Silixa iDAS and a Sintela Onyx interrogator, hereafter referred to as iDAS and Onyx. The iDAS was only deployed at Double Crater Flow as the site allowed access to a generator, and the iDAS requires more power than the Onyx. Table 1 describes the acquisition parameters used for each type of seismic equipment. For the DAS systems, there are two spatial sampling parameters. Gauge length is the length of the fiber optic cable over which the DAS interrogator determines the average ε. This value is the minimum spatial wavelength resolvable by the DAS system, or the Nyquist wavelength. Distributed acoustic sensing systems can also over sample spatially, yielding more channels than independent gauge lengths along the fiber. For the geophones and TC, the geophone or shot spacing provides the spatial Nyquist wavelength and gives the minimum resolvable seismic wavelength for these systems. Due to the inherent constraints of the DAS systems configurations and settings, we could not perfectly align gauge lengths/channel spacings and minimum resolvable spatial wavelengths between the different seismic equipment used. Tap tests and shots were used to determine the DAS channel locations and their relative positions to the geophones and TC. Gauge length choices tended to smooth out signals between channels, but we are likely accurate to within a channel spacing for a given location. For most analyses the sampling rates and times were synchronized via downsampling where necessary using a low pass FIR filter to prevent temporal aliasing. Records were interpolated to a common temporal sampling of 200 Hz. The DAS records exhibited sharp jumps after shots, likely due to the large ε or clipping of large ε signals on the fiber. These jumps were removed by applying a median filter of 25 samples in time for each spatial location to the DAS records. All the data were demeaned and detrended for each recording period, and we applied a 4th order Butterworth bandpass filter from 1 to 200 Hz to the TC data.

**Table 1:** Acquisition Parameters

Sintela onyx interrogator	
Gauge length	4.79 m^a^
Channel spacing	1.6 m
Sampling rate	1,000 Hz (Downsampled from internal 40 kHz rate)
Silixa iDAS	
Gauge length	10 m^b^
Channel spacing	1 m
Sampling rate	1,000 Hz
Nanometrics trillium compact	
Sampling rate	200 Hz
Geometrics geode seismograph/4.5 Hz geophone	
Sampling rate	8,000 Hz

^a^ This was the minimum possible value for this system.

^b^ Fixed by the manufacturer.

A primary motivation of this work is to understand the relative merits of DAS in comparison to traditional seismic equipment for determining shallow structure. The TCs and geophones both have advantages and disadvantages relative to DAS systems. Distributed acoustic sensing systems record ε or strain rate on the fiber optic cable, which is different than most standard seismic equipment which records ground accelerations or velocities which can be converted to displacement by integration. So a response function with units of m/ε or m/strain rate is required to relate the recordings of DAS to ground displacement. The geophones measure vertical ground velocity. We used 24, 14 Hz geophones at Double Crater Flow and 24 4.5 Hz geophones at Lava River Cave. The change was related to availability, and both are sensitive to 1–1,000 Hz. Although geophones are relatively cheap, they need to be fixed into the ground, require multiple wired connections, and are heavy in comparison to fiber optic cables; all of these aspects are time consuming, and/or present operational challenges on extraterrestrial bodies. The TCs record 3 components of ground velocity particle motion and have a flat and well‐calibrated response from ∼0.008–100 Hz, allowing us to fully evaluate the response of the DAS system to ground motions. The TCs are more expensive than individual geophones, and therefore provide less spatial granularity on a shot‐by‐shot basis.

The active source experiments consisted of shots generated by manually driving a 4.5 kg hammer from ∼2 m height onto an 0.5 inch thickness aluminum metal plate. Hammer shots were performed at both study sites. At each site, for consistency the same person was used to generate the shots. At Lava River Cave, shots were performed once every 1 m along line 2 starting at 60 m through 83 m. On line 3, shots were performed every 1 m starting at 0 m. At Double Crater Flow, shots were performed every 2 m from 0 to 50 m starting at 0 m along line 4. The shots were all performed along the same side of the fiber, offset from it by a small amount, <10 cm. To compare DAS recordings to geophones and the broadband data, we used recordings of the same shot for DAS and geophone data located at a range of distances from the shot. To compare to the TC data, we constructed common receiver gathers of several shots at different locations/distances from the TC. The TC can be treated as the shot location and the shot locations as the recording stations if Betti's reciprocity approximately holds (Aki & Richards, 2002). Finally, we make comparisons between all three systems where shot locations are co‐located with the TCs.

The DAS and TC recorded continuously for several hours at each site during the 2 days of the experiment. Unfortunately, there were no earthquake events during our recording period, so we could not assess the surface deployed DAS for passive source work.

Power consumption for the DAS interrogators was low enough that portable power sources could be employed for the hours‐long durations of the experiments. We used 100 AmpH batteries to run the Onyx system at Lava River Cave for the duration of the work (∼4 hr). At Double Crater Flow, we used the same batteries to power the Onyx and iDAS for ∼1.5 hr, before the batteries were exhausted and we needed to switch to generator power. The change to generator power impacted the signal‐to‐noise ratio in the limited frequency range of the generator, but it did not substantially affect our analysis because of the considerable distance between the generator and DAS cable (Figure 1, Figure S1 in Supporting Information S1). The change to a generator was required both because we were using two interrogators and also because the iDAS requires more power than the Onyx; the Onyx typically uses ∼100 W, and the iDAS typically uses ∼300 W.

### 2.2 Analyses of DAS, Geophone and Broadband Data

We performed standard seismic analyses on individual shot sections and gathers to examine the efficacy of DAS for these analyses. To start, we picked first arrivals to identify refractions using geophone and DAS data, which provided information on the P‐wave velocity (Vp) structure. For each data set we generated a 1‐D Vp structure using the slope‐intercept method to constrain the shallowest layer thickness and velocity (Telford et al., 1990) for line 4 with shots at −20, 0, 46, and 66 m. We then used 1‐D travel time calculations using a fast marching algorithm (Sethian, 1999) to invert the data from 4 shots for the Vp structure of the upper 30 m. In addition, we performed multichannel analysis of surface waves (MASW) on the gathers to estimate Rayleigh wave dispersion on the shot sections and gathers. We used phase velocity‐frequency analysis to measure the Rayleigh wave dispersion. We correlated plane waves with a range of wavelengths (1–200 m) over the frequency range of interest (5–100 Hz), each corresponding to a phase velocity and frequency, with the Fourier Transform of the array data at the specified frequency. We determined the phase velocity of the different Rayleigh wave modes by picking the peaks of high correlation ridges in the velocity‐frequency maps. We inverted the change in phase velocity with frequency (dispersion) of the first three modes observed by the geophones and DAS independently to estimate the shear‐wave velocity (Vs) of the upper 30 m of meters in the region. We used the information from the Vp structure as the starting model for inverting the surface wave modes data. We used the Computer Programs in Seismology software suite to model the surface wave dispersion (Herrmann, 2013).

We calculated the empirical response function of the DAS ε recordings using the ground velocity measurement from the radial (R), transverse (T), and vertical (Z) components of the TCs. We did not calculate responses for DAS relative to the vertical geophones and instead focused on the multi‐component contributions to the DAS signals. The broadband seismometers were instrument response corrected to ground displacement. The broadband instrument horizontal components were rotated into R and T components, corresponding to the DAS fiber orientation. R is oriented in line with the azimuths of lines 2, 3 and 4, and T is perpendicular to the azimuth for the lines but parallel to the x‐line for line 4 (Figure 1). We selected time windows containing shots for analysis and used traces of the DAS inline and x‐line at Double Crater Flow nearest the broadband seismometers to calculate the response function. The empirical response function (ER) as a function of frequency f, ER(f) was calculated following Harmon et al. (2022):

(1) $$ER\left(f\right)=\frac{C_{DH}\left(f\right)}{C_{DD}\left(f\right)},$$

where C_DH_ indicates the cross spectral estimate between the DAS subscript D, and a given broadband component subscript H, and C_DD_ is the auto spectral estimate of the DAS (Bendat & Piersol, 2000). This is different from previous work (Lindsey et al., 2020; Paitz et al., 2020; Wang et al., 2018), which corrected the DAS channel data to velocity via frequency domain multiplication by phase velocity. We prefer this approach as it does not require any assumptions about velocity structure and is relatively straightforward. We also used spectral coherence *γ*(*f*) to assess the applicability of the empirical response:

(2) $$\gamma \left(f\right)=\frac{{\left|C_{DH}\left(f\right)\right|}^{2}}{\left|C_{HH}\left(f\right)\right|\left|C_{DD}\left(f\right)\right|},$$

where *C*_*HH*_ was the autospectra of the broadband component (Bendat & Piersol, 2000). Spectral coherence close the value of 1 at a given frequency indicates a reliable response function, while values lower that 0.6 are likely unreliable (Bendat & Piersol, 2000).

## 3 Results

**Figure 3:**
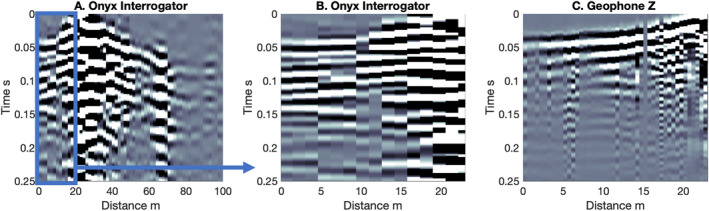
Shot comparison between recording methods along line 3 at Lava River Cave. The hammer shot is located at 23 m. (a) Onyx Distributed acoustic sensing (DAS) shot record 0–84 m along the line. Box indicates region of zoom (b) Zoom of the Onyx DAS from 0 to 23 m, corresponding to (c) vertical geophone recording from 0 to 23 m. There were not enough shots to generate a shot section from the TC at 0 m on line 3 at Lava River Cave.

**Figure 4:**
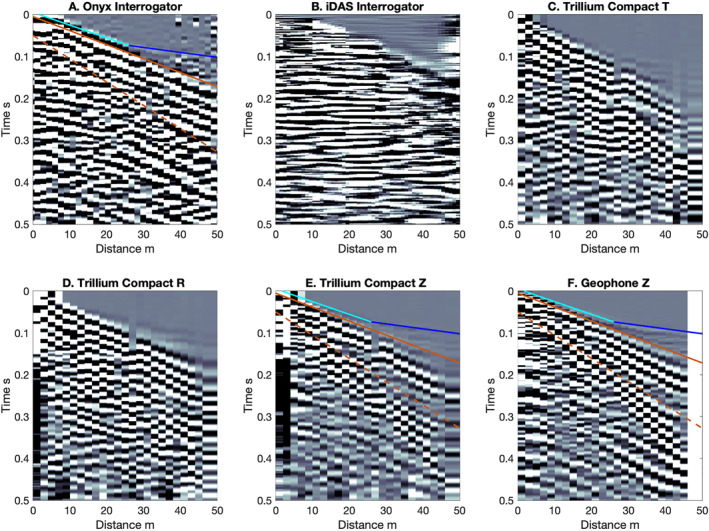
Comparison of shot at 0 m on line 4 at Double Crater Flow, (a–f) Onyx, iDAS, TC T, R, Z components and Z‐component geophones. Cyan and blue lines show moveouts of 330 and 850 m/s respectively. Orange solid and dashed show 300 m/s and 180 m/s moveouts, respectively. The cross over point is at 26 m.

We compare Onyx and iDAS interrogator shot recordings to TC gathers and vertical component geophones at the Lava River Cave site and Double Crater Flow (Figures 3 and 4). The three‐component common TC receiver gathers allow us to visually assess similarity between the different components of ground motion and the DAS recordings. The geophone and TC records are shown in units of velocity, and the Onyx and iDAS are in units of strain rate.

The Onyx interrogator, which recorded at both sites, records many of the phases observed in the traditional seismic equipment. At Lava River Cave, the low frequency arrival visible in the geophone data agrees well with the first arrival visible in the Onyx DAS record (Figure 3). A shot section is not shown for the TC at 0 m of line 3, as there were not enough shots generated at Lava River Cave. For a shot at 0 m on line 4 at Double Crater Flow, there is good agreement between the Onyx, TC, and geophone recordings on the slope at close distances (330 m/s, cyan line Figures 4a, 4e, and 4f) and the location of the change in slope (26 m; intersection of blue and cyan lines in Figures 4a, 4e, and 4f). The slopes of the first arrivals at >26 m (850 m/s, blue line Figures 4a, 4e, and 4f) also agree. In addition, the higher amplitude and lower frequency energy, later arrival of Rayleigh wave energy at 30–59 m (∼300 m/s), is also clearly visible in the Onyx data, the geophone, and TC Z and R component (orange lines). The Onyx data does not show a distinct arrival with a move out of 180 m/s (orange solid and dashed lines), which makes it qualitatively look more similar to the R component than the Z component common receiver gather. Overall, the DAS first arrivals observed at both sites agree visually with those observed on the geophones and/or broadband common receiver gathers (R and Z components).

The iDAS interrogator was only used at Double Crater Flow. The results did not compare favorably with the seismic equipment (Figure 4b). The first arrival at 330 m/s is visible on the iDAS record; however, the phases after the first motions do not exhibit a clear moveout, rather they arrive at the same time. In other words, the phases are flat in time.

**Figure 5:**
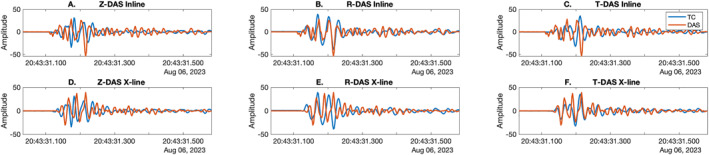
Comparisons of inline (a–c) and x‐line (d–f) Onyx traces (red) and TC (blue) Z, R, and T components (a–c, d–f) at 25 m for shot at 38 m at Double Crater Flow. Components are indicated for each panel.

**Figure 6:**
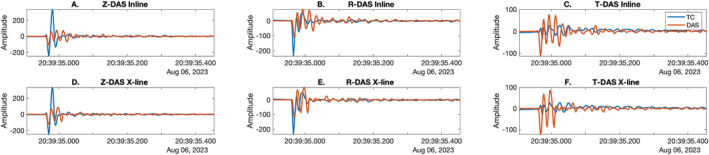
Comparisons of inline (a–c) and x‐line (d–f) Onyx traces (red) and TC (blue) Z, R and T components (a–c, d–f) at 25 m for shot at 24 m at Double Crater Flow. Components are indicated for each panel.

Comparison of individual traces highlights reasonable agreement between phases observed on the inline and x‐line Onyx DAS records at 25 m on line 4 at Double Crater Flow with the TC's R and T components (after correcting the TC for instrument response and scaling by 1 × 10^10^, owing to differences in units, for comparison purposes; Figures 5 and 6). For example, for a shot located at 38 m, the inline fiber channel nearest the TC located at 25 m shows good agreement with the R component for the first 0.14 s of the trace with a correlation coefficient of 0.47, while the transverse component is out of phase with a correlation coefficient of 0.20 (Figure 5). The TC Z component is 180° out of phase (negative) with the DAS record and has a correlation coefficient of −0.51. The first arrival visible in the inline fiber channel has a slightly different character than the first arrival visible on the Z and R component, which may be related to median filtering artifacts. For the x‐line DAS channel, the T component is in phase for the first 0.14 s of the first arrival with a correlation coefficient of 0.91, while the Z component and R component are less similar with correlation coefficients of −0.28 and 0.56 respectively. Specifically, the first arrival is visibly delayed in the Z and R components by ∼0.01 s. For a shot located at 24 m, near the crossing point of the fiber, relative to the TC at 25 m, the Z component is in phase with the inline and x‐lines for the first 0.08 s of the record with a correlation coefficient of 0.54 and 0.74, respectively. The R component for the inline and x‐line over the same time period has correlation coefficients of 0.73 and 0.65, respectively (Figure 6). The T component shows an opposite sign to the DAS component for both the inline and x‐line for the first motion with the lowest correlation coefficients of −0.12 and −0.02, respectively, for the same time period.

**Figure 7:**
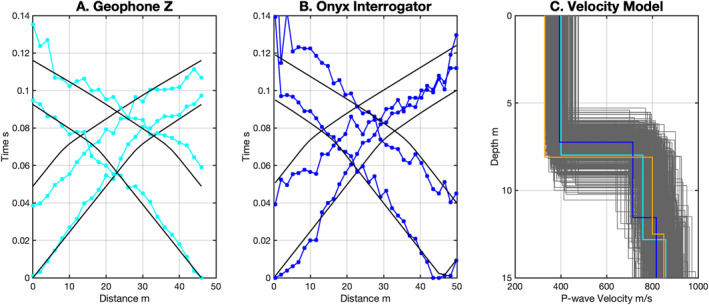
Comparison of P‐wave traveltimes for shots at −20, 0 46 and 66 m for (a) geophone, (b) Onyx interrogator at Double Crater Flow, Line 3. (c) Shows the velocity models determined from each set of travel times, for geophones (blue) and Onyx Interrogator (cyan). The starting model is for both inversions is shown in orange, and the range of acceptable models is shown in gray background models. Black lines in panels (a, b) indicate the predicted arrival times for respective models in panel (c).

Travel time picks for first arrivals for shots located at −20, 0, 46 and 66 m on line 4 at Lava River Cave show good general agreement between the vertical geophones and the Onyx interrogator (Figures 7a vs. 7b). There is more scatter visible in the travel time picks for the Onyx interrogator, exhibiting typically <0.005 s but up to 0.02 s differences between adjacent channels in some cases. This was due to higher noise on the Onyx DAS and difficulty identifying the coherent phase between traces (Figure S2 in Supporting Information S1), in particular, for the shot at −22 m. This shot was performed after the generator was turned on. More coherent noise is visible in this section prior to the seismic arrivals. The slownesses observed between the geophones and the Onyx interrogator are comparable. P‐wave velocity models derived by inversion independently from the either data set (Figure 7c) are similar in value in terms of layer thickness and velocity structure. The predicted fits to the data are within a nominal 0.02 picking error for both data sets. The suite of models that satisfy both the DAS and geophone arrivals within a nominal 0.02 s error are shown in gray in the background and indicate a range of velocities layer thickness for our 3‐layer model.

**Figure 8:**
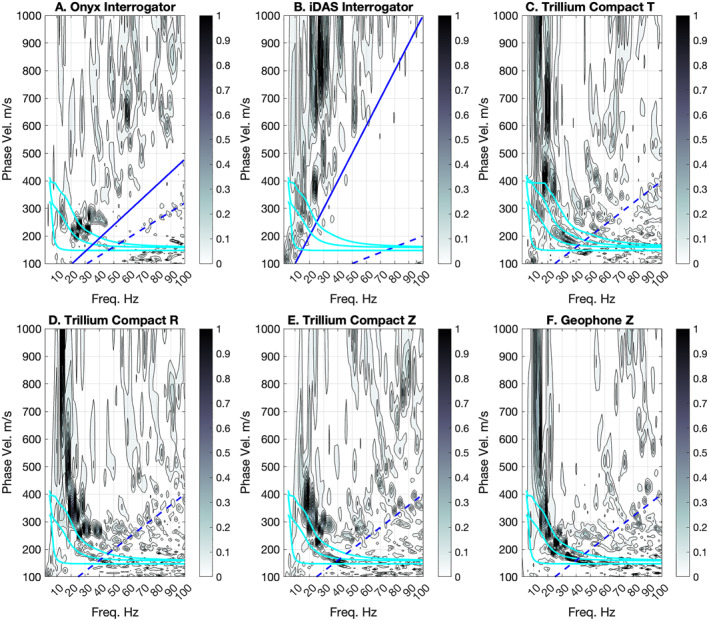
Surface wave multichannel analysis of surface waves plots (a) Onyx, (b) iDAS, (c) TC T, (d) TC R, (e) TC Z, (f) and geophones are shown. Solid blue line shows the gauge length (internal spatial averaging of Distributed acoustic sensing) and the dashed blue shows the channel spacing spatial Nyquist. Cyan lines show the predicted dispersion for the first 3 Rayleigh wave modes for the best fit forward velocity model (Figure 9). The predicted dispersion is the same in all panels based on the best‐fit model from the geophone vertical data, but the energy visible in panel (c) corresponds to the Love wave dispersion.

The MASW analysis comparisons between the Onyx and iDAS systems and the TC and geophone data show that the DAS systems recover the lower frequency components of the Rayleigh wave modes (Figure 8, Figure S3 in Supporting Information S1). The Z and R component TC, geophone, and Onyx exhibit multiple mode Rayleigh wave dispersion, visible as curving areas of high normalized energy between 10–50 Hz with magnitudes >0.4 (Figure 8). The performance of the iDAS is poor in these frequencies and does not appear to exhibit Rayleigh wave mode dispersion, likely due to the fixed gauge length of the iDAS system. The traditional seismic equipment exhibits dispersion down to very low velocities of 150 m/s. At phase‐velocity‐frequencies below the gauge length wavelength (blue line Figure 8a) there is little to no energy visible in the Onyx record. The Onyx dispersion looks more similar to the R and Z component analysis, with peaks in energy visible between 200 and 300 m/s at 20–30 Hz. The Onyx does not have much energy above 300 m/s between 10–40 Hz, although this energy is visible on the R and Z components of the TC and geophones. This is likely because the fiber length that was deployed was too short to resolve larger wavelengths. Dispersion is visible in the T component TC from Love waves.

**Figure 9:**
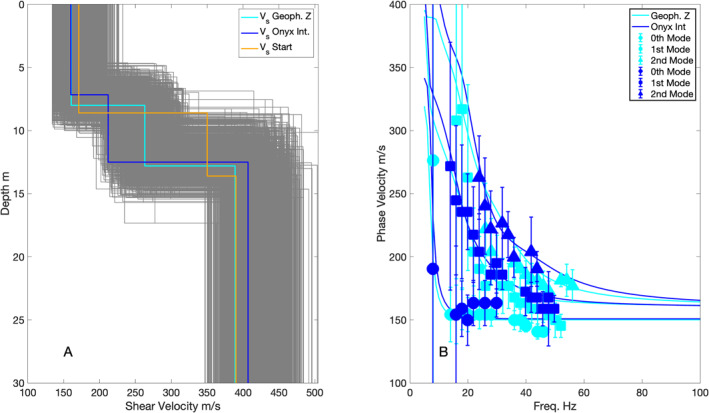
Comparison of multichannel analysis of surface waves (MASW) inversion Vs velocity model results for vertical geophone data (cyan) and the Onyx interrogator (blue) (a), and observed modes (b) (cyan‐geophone data, blue—Onyx data) and predicted dispersion for the first three modes (blue and cyan for respective models). The starting model for both inversions is shown in orange, and the range of acceptable models is shown in gray background models. Detailed MASW picks are shown Figure S3 in Supporting Information S1.

We plot our inverted Vs model for Line four of Double Crater Flow (Figure 9a) based on the refraction first arrivals (Figure 7) and the dispersion from MASW (Figure 8). The dispersion measurements between the geophone data and the Onyx have overlapping errorbars at most frequencies where picks could reliably be made. The Vp in the best fit models ranges from 394 to 401 m/s in the shallowest 7.2–8.0 m thick layer to 817–858 m/s in the deepest layer down to 30 m (Figure 7). Vs in the best fit models ranges from 160 to 161 m/s in the shallowest layer to 389–406 m/s at 30 m depth (Figure 9; Table 2). For reference we plot the predicted dispersion curves for the first three Rayleigh wave modes which show a good agreement with the observed dispersion for the Z component TC, R component TC, geophone, and Onyx data (Figures 8 and 9). The T component dispersion visible between 150 and 220 m/s at 20–40 Hz lies in between the Rayleigh wave mode dispersion curves (cyan lines, Figure 8c).

**Table 2:** 1‐D Velocity Model for Line Four

Depth *m* geophone/onyx/thickness range	Vp m/s geophone/onyx/range	Vs m/s geophone/onyx/range
0–8.0 (0–7.2)/5.2–10.5	401/394/330‐484	161/160/134‐231
8.0–12.8/8.2–12.5/1.3–7.6	758/714/640‐926	263/211/182‐286
>12.8/12.5/8–17.3	858/817/713‐987	389/406/355‐504

The MASW plots (Figure 8) highlight some limitations of the DAS systems imposed by gauge length selections possible from the manufacturer (Onyx) or those that are fixed (iDAS) and geophone/shot spacings which determine the spatial sampling. The phase velocity—frequency plots are based on measuring the wavelengths of the surface waves recorded across the array in the Fourier spatial domain. We plot lines of constant wavelength in the phase‐velocity—frequency space for the gauge length (solid blue) and spatial sampling Nyquist (2 x channel spacing, dashed blue). For the Onyx and iDAS, very little energy is observed below the gauge length line, which is to be expected given that it is a spatial filter. In comparison, the traditional seismic equipment shows some evidence for spatial aliasing, specifically patchy higher energy areas, below the Nyquist line.

**Figure 10:**
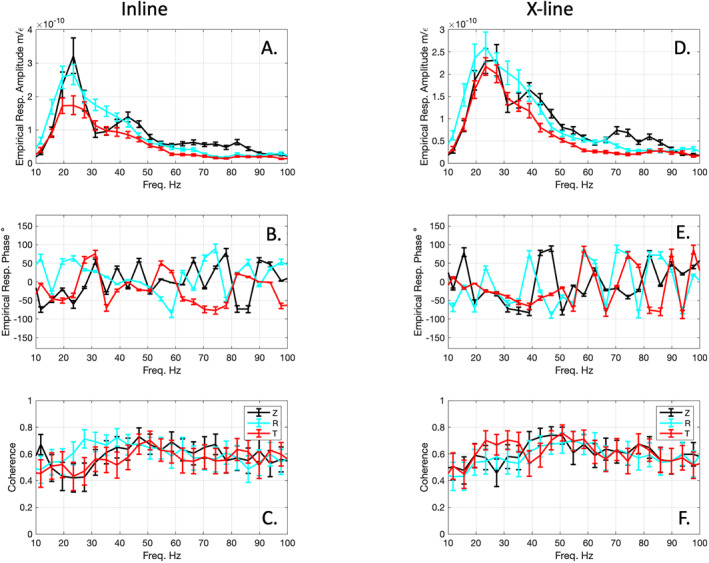
Empirical response of Onyx DAS determined from the TC Z (black), TC R (cyan), and TC T (red) components. Left panels are for inline fiber and right panels are for x‐line fiber to TC. Top row (a, d) shows the amplitude response, middle row shows phase response (b, e) and bottom row shows coherence values (c, f).

Empirical response functions generated by stacking the responses from 25 shots for the inline and x‐line Onyx DAS components calculated relative to the three TC components highlight the horizontal sensitivity of the surface deployed DAS (Figure 10). For the inline DAS channel, the amplitude response for all three components is similar, with a peak centered between 20–30 Hz and ranging from 0.9–2.5 × 10 ^−10^ m/ε (Figure 10a). This peak is roughly the center of the energy generated by each shot. The response decreases monotonically away from this peak at both higher and lower frequencies. The phase response is not stable for the Z and T components, rapidly changing with increasing frequency by 10s of degrees (Figure 10B). The R component exhibits a linearly changing phase angle from 55° to −5° between 20 and 50 Hz. The coherence between the DAS and R component is also the highest (∼0.7) in the 20–50 Hz range suggesting the DAS channel is recording primarily the horizontal inline radial energy (Figure 10c). For the x‐line similar patterns can be observed in terms of the peaked amplitude response and phase behavior for the T component, that is, 1.6–2.3 × 10 ^−10^ m/ε and phase −5° to −66° from 20–50 Hz (Figures 10d and 10e). The coherence is more band limited for the T component being above 0.7 from ∼20–40 Hz (Figure 10f). At higher frequencies the R and Z components are more coherent with the x‐line DAS channel. We speculate that the x‐line DAS sensitivity to the Z and R component may be owing to the inclusion of near‐field shot locations, where body and surface waves arrive nearly simultaneously.

**Figure 11:**
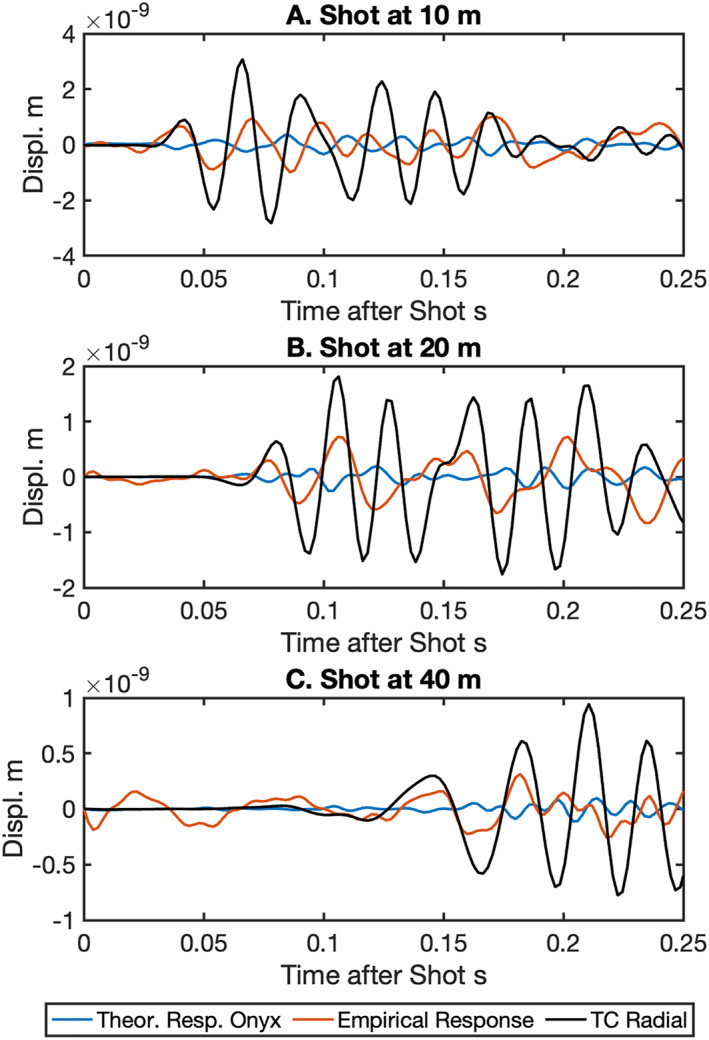
Cross‐validation of empirical response between Radial component of TC and Distributed acoustic sensing (DAS) channels converted to displacement. We use shots at 10, 20 and 30 m from the TC located at 0 m, and a shot at 0 m and DAS channels located at 10, 20 and 30 m. We convert the DAS records using the Sintela parameters 1.08 × 10^−11^ m Hz^−.5^ (blue line) and our derived empirical response functions (orange) in comparison the TC (black line).

We perform a cross‐validation test of our empirical response function to assess its robustness. For the test we use the radial component of the TC located at 0 m on the Double Crater Flow line 4 as the ground displacement reference (black line, Figure 11), which is different than the TC used to calculate the empirical response functions at 24 m at the x‐line. We compare the Onyx Interrogator channel recordings at 10, 20 and 40 m of the shot at 0 m (orange lines, Figure 11), to the TC recordings of the shots at 10, 20 and 40 m. The DAS recordings are converted to displacement using the empirical response functions for the radial component (Figure 10) as well as the predicted ground displacement using Sintela's conversion specified for a bare fiber of 1.08 × 10^−11^ m Hz^−0.5^ applied in the spectral domain (blue lines, Figure 11). Neither response function brings the entirety of the records into phase with the TC, although the empirical response appears to bring the largest first arrivals into phase. Correlation coefficients for the first phase ranges from 0.4 to 0.9 for the empirical response functions, while the Sintela conversion was −0.3 to −0.8. The phase mismatch becomes more evident after the first few cycles of the waveform. Sintela's conversion does not generate a phase shift but does bring the DAS records within 10%–20% of the amplitude of the ground displacement recorded by the TC. The empirical response gets the amplitude closer to the TC, within 30%–40% where the two signals are in phase.

## 4 Discussion

The different performances of the DAS systems is related to the fixed gauge length of the iDAS system used here. The Onyx system has several choices for gauge length, while the iDAS had a fixed gauge length of 10 m which appears to result in severe spatial aliasing. This is likely the reason that the Onyx compares better with the traditional seismic equipment. Testing of larger gauge lengths with the Onyx system showed similar spatial aliasing issues to the iDAS (not shown). The iDAS may perform better for longer survey lines than the ones performed here, and has been demonstrated to be effective for active source work over several hundred meters or more with stacking of repeated shots (Daley et al., 2016; Dou et al., 2017; Jousset et al., 2018), although this was beyond the scope of the GEODES experiment. Therefore, we focus on the Onyx results.

The DAS systems primarily record horizontal component information, based on the highest observed coherence in the empirical response functions of the inline DAS with R component and the x‐line DAS with the T component (Figures 10c–10f). This suggests greatest sensitivity to ε in the inline fiber direction. The DAS system does seem to capture a small amount of vertical motion, based on the correlation coefficients between the Z component at the Onyx DAS for some of the shots. Slight topography may give the DAS a small vertical sensitivity, although the topographic slopes were probably <2° within our survey lines. The TC was leveled to within 0.5°. So likely the R and T components represent purely horizontal ground motions. The DAS sensitivity is in good agreement with theoretical predictions for seismic phases impinging on a fiber, which suggests P‐wave and Rayleigh wave phases at low angles to the fiber axis will generate the largest ε, while S‐waves and Love waves will generate the greatest ε at ∼45° (Kuvshinov, 2016). Although DAS primarily only records 1 component of motion, DAS data can be used in conjunction with traditional seismic instruments to perform analyses that require multiple components of motion, for example, receiver functions (Yu et al., 2019).

In addition, analyses of just the 1 component of motion from the DAS provided structural information that agreed reasonably well with that from traditional seismic equipment. For example, first arrivals are visible in the Onyx system and agree well with the first arrivals visible in both geophone shot sections and TC common receiver gathers (Figure 4, blue/cyan lines). Refraction analyses applied to these arrivals successfully recovered near surface Vp very similar to that from geophones. Surface wave information was partially recovered from the Onyx DAS system at wavelengths longer than the gauge length and showed general agreement with the modes recorded by the traditional seismic equipment in the lower frequency range (Figures 4 and 7). The mode dispersion was successfully inverted for near surface *V* *s* that was very similar to that determined by inverting the geophone dispersion observations.

We restricted our analysis to 1‐D velocity structure. A basic interpretation of our 1‐D structure is that the loose and unconsolidated regolith extends to the bottom of the first layer of our model, 8.1 m, which is roughly consistent with the thickness of Double Crater lava flow observed nearby in the field. Greater consolidation within the regolith layer of an older flow is likely required to explain 714/758 km/s *V*_*P*_ between 8.1 and 13 m, and the deepest layer likely corresponds to a bedrock layer. The observed arrival times are systematically different than the predictions for a simple 1‐D model, for instance, arriving earlier for shots on one side of the line (0–12 m) (Figure 7a). This could be evidence of 2‐D structure such as a dipping layer. However, the scatter in the DAS data is too great to warrant further analysis, and we did not perform 2‐D imaging with the DAS data to image the depths of the lava tube or to the base of the cinder, even if these were the broader targets of the broader GEODES field program. The 1‐D structure can be used to improve imaging of deeper structures in combination with other methodologies. The 2‐D imaging is a topic of future work, which could potentially be improved by performing and stacking multiple shots in each location.

The DAS systems do have limitations when compared with the traditional seismic systems. In the shot sections, Onyx DAS records similar phases as the TC and geophones but with notable differences in signal‐to‐noise ratio and frequency content; the visual comparison of the traces makes this clear. The greater amount of noise, evidenced by the speckle in the time periods before the P‐wave in Figure 4 and Figure S2 in Supporting Information S1, in the DAS data affected our ability to precisely pick P‐wave arrivals for refraction work, resulting in greater scatter in the travel time picks. This is mainly because the experiment was primary designed to perform imaging with the geophones, which do not necessarily require stacking of multiple shots; although this seemed to have been less ideal for the DAS data. However, previous work has demonstrated that stacking multiple shots can reduce noise in data from DAS (Harmon et al., 2022). In addition, more sophisticated methodologies based on machine learning might be employed to aid in picking DAS data (e.g., Zhu et al., 2023).

In addition, the DAS systems are band limited by the gauge length, which limits the minimum seismic wavelength that can be recorded by the system. Seismic energy is recorded with the phase velocity dependent fiber sensitivity characteristic. This is well known (Daley et al., 2013, 2016; Lindsey et al., 2020), and our results demonstrate this clearly in the phase‐velocity‐frequency plots (Figure 7, Figure S3 in Supporting Information S1). The use of shorter gauge lengths in future work will help to ameliorate this issue for high resolution work, with the caveat that some prior knowledge of the lowest velocities or shortest wavelengths, which requires some estimate of near surface velocities, is needed to guide the choice. In addition, there is a tradeoff between using a shorter gauge length and instrument noise floor, that is, shorter spatial averaging results in higher noise levels.

The empirical response functions also highlight the need for further testing. The amplitude response of the DAS systems to all channels appears to be stable, with the inline and x‐line DAS producing similar shaped responses of a similar magnitude (1.5–3.0 × 10^−10^ m/ε) for the Onyx system. The phase response, however, was more variable and not 0° phase (in phase) and flat as reported by previous work (Lindsey et al., 2020; Paitz et al., 2020). Unfortunately, we were not able to make comparisons between weighted fiber and the TC to determine if there was a similar phase shift due to the coupling in the empirical responses. Our cross‐validation of the empirical response demonstrates that the amplitude of ground motion is recovered within 30%–40% of the ground displacements recorded on the TC, but the phase is only partially recovered. This could be due to imperfections in the TCs response, processing artifacts, complex surface velocity/attenuation effects and/or imperfect coupling of the fiber.

The response of the TCs is well known, and empirical responses using different TCs were very similar to the one shown here and the TCs seismograms were consistent with each other. The processing of the DAS could play a role in the differences in the signal recordings. The median filter was required to remove jumps to avoid cycle skipping in the interferometry. Better processing to avoid these issues needs to be developed for DAS. Our approach for determining the response is different from in previous work (Lindsey et al., 2020), but this difference will not result in a frequency dependent phase shift. Previous work, also observed systematic phase shifts at some frequency bands (Paitz et al., 2020), so it is unlikely that our choice of response modeling is the cause of the phase shift. Sharp lateral changes such as sediment to solid rock in the near surface elastic properties (over less than a gauge length) as seen at Lava River Cave could result in differences between the DAS records and those of the TCs. This would be less of an issue in previous work that was performed at lower frequency (Paitz et al., 2020). However, this may not be a universal reason since we did not observe such sharp changes at Double Crater Flow.

Imperfect coupling is the most likely cause, since our fibers were buried very shallow in comparison to previous work using dark cables (Dou et al., 2017; Lindsey et al., 2020; Lindsey & Martin, 2021; Yang et al., 2022; Zhan, 2019). Poor coupling can act like attenuation and cause frequency dependent phase shifts especially if there is physical sliding between the cable and the substrate during the propagation of seismic waves (Aki & Richards, 2002; Reinsch et al., 2017). The low‐density cinder/ash and low mass of the fiber may have led to a low effective frictional coupling of the fiber. Future work will need to develop optimal coupling and site assessments of lateral structure changes.

Our choice of fiber was economical, but for long term durability in the environment, armored or ruggedized cables will need to be used. This will not only add weight but also increase the labor required for deployment. Automation or robotic partnerships may facilitate laying the cable efficiently over complex terrain. Future work will need to combine different aspects of this previous work in a systematic fashion to develop optimal fiber construction, coupling strategies for different substrates and sensitivity for use in planetary science for near surface geophysics.

The findings of our work build on previous work showing surface deployment of DAS systems is effective. The earlier works have focused on different aspects of fiber choice, coupling strategy and substrate coupling. For instance, we used only one fiber type, but one study examined the effect of fiber optic cable choice weighted down to the ground on the fidelity of DAS recordings to vertical geophones (Spikes et al., 2019). The DAS recordings on the standard fiber types were similar to the geophone shot sections, but with significant differences, likely due to DAS records being dominated by horizontal ground ε (Spikes et al., 2019). Here we examined shallow burial and weighting, while another study showed the effectiveness of pinning as a coupling strategy for surface deployment and the horizontal component nature of surface DAS recordings (Harmon et al., 2022). We examined two new substrates, in comparison to previous work which examined the effect of surface deployment on different substrates (concrete, grass, and snow) on coupling for use in hazard monitoring (Mjehovich et al., 2023). This work and previous work has demonstrated the effectiveness of fiber in diverse substrates (Mjehovich et al., 2023). Our work demonstrates weighting and shallow trenching are viable mechanisms for adapting surface deployment strategies to the substrate.

## 5 Conclusions

We successfully deployed DAS at two lunar analogue field sites using two different coupling techniques in response to the terrain. Both sites presented operational challenges that could reasonably be present for a DAS deployment on the Moon. At Lava River Cave, the terrain was covered by boulders and rocks for many parts of the lines, but it was relatively straight forward to deploy in this terrain with weighting and placing the fiber in and around large obstacles. We supplemented our sandbags with rocks from the landscape for additional weights. The weighting coupling strategy was effective and practical. However, the sand bags needed to be carried in by hand. Pinning of the fiber to the ground might be more optimal in this terrain for planetary purposes given the weight of the sandbags, and readily available regolith for burial or weighting the cable. Although we did not evaluate the response and coherence because there was no x‐line and a 3 component evaluation was not possible, previous work has shown the effectiveness of pinning (Harmon et al., 2022). At Double Crater Flow the cinder substrate of the terrain was amenable to shallow burial with excavation of a small trench (<10 cm deep) with simple hand tools. This was also an effective coupling strategy, but more labor intensive than weighting or pinning in this case but required less mass to couple the cable. Burying the fiber added approximately 1 hr to the deployment with two people, but the additional time required would be reduced with the selection and development of more advanced deployment tools and techniques. For comparison, deploying 4 TC took a similar about of time, while the geophones were deployed in ∼30 min. Burial also has the advantage of reducing interactions of the fiber with wind, local vegetation, and disturbance by the team tripping or bumping the cable (incidentally a long cable posed a tripping hazard to the Apollo astronauts). Ideally, future work will compare responses between the two DAS coupling strategies quantitatively. This was not tested in this experiment due to limited time. Finally, other planetary terrains, such as scree, ice, etc. Will require development of more specialized deployment and coupling techniques.

In conclusion, surface deployed DAS presents a potentially powerful tool for near‐surface geophysics on planetary bodies. Our field tests at the lunar analogue sites in the San Francisco volcanic field demonstrated their potential on the moon, effectively recording seismic shots in two different landscapes with complex near surface heterogeneity. The cables themselves were relatively easy and rapid to deploy in comparison to the traditional seismic array that required heavier cables and installation of instruments, suggesting the DAS would simplify a seismic system that could be deployed on crewed or robotic missions. Fiber requires laying only a single cable with one connection to the data recording system. Fiber is also simple to repair via fusion splicing relative to more sophisticated seismic equipment such as land streamers or geophone strings. We have demonstrated a DAS system can be used to perform traditional seismic analysis, with the caveat that the DAS is primarily sensitive to horizontal ground ε and gauge length is a limitation on the shortest resolvable seismic wavelengths. Consideration of the possible material properties of the subsurface against choices of gauges length is crucial for resolving structural information at the desired scales. More work is required to develop these systems for future missions, such as developing optimal field deployment and acquisition techniques, software, and ruggedization. Future experiments also need to be performed at the extreme conditions of planetary bodies where large fluctuations in temperature and high radiation level may impact the fiber and interrogator. In addition, weighting‐based coupling strategies developed under Earth's gravity conditions may be less effective on smaller planetary bodies where the gravitational attraction is less. Distributed acoustic sensing systems can potentially provide an excellent complement to other geophysical techniques for near‐surface applications on planetary bodies. This is especially true since fiber optic cabling will likely be deployed along planetary surfaces anyway, to connect other scientific equipment and for communications purposes.

## Supporting information

Supporting Information S1

## Data Availability

The DAS time series data used in the study are available at pure.soton.ac.uk (Harmon et al., 2024) with open access creative commons license.
