# Supplementary material for: Distributed Acoustic Sensing for Future Planetary Applications: Initial Results From the San Francisco Volcanic Field, a Lunar Analogue
Source: Earth Space Sci. 2024 Nov 27;11(12):e2024EA003640. doi: 10.1029/2024EA003640 (PMC11602534; doi:10.1029/2024EA003640)
Supplement: Supplementary file 1 — Supporting Information S1 [file ESS2-11-0-s001.docx]

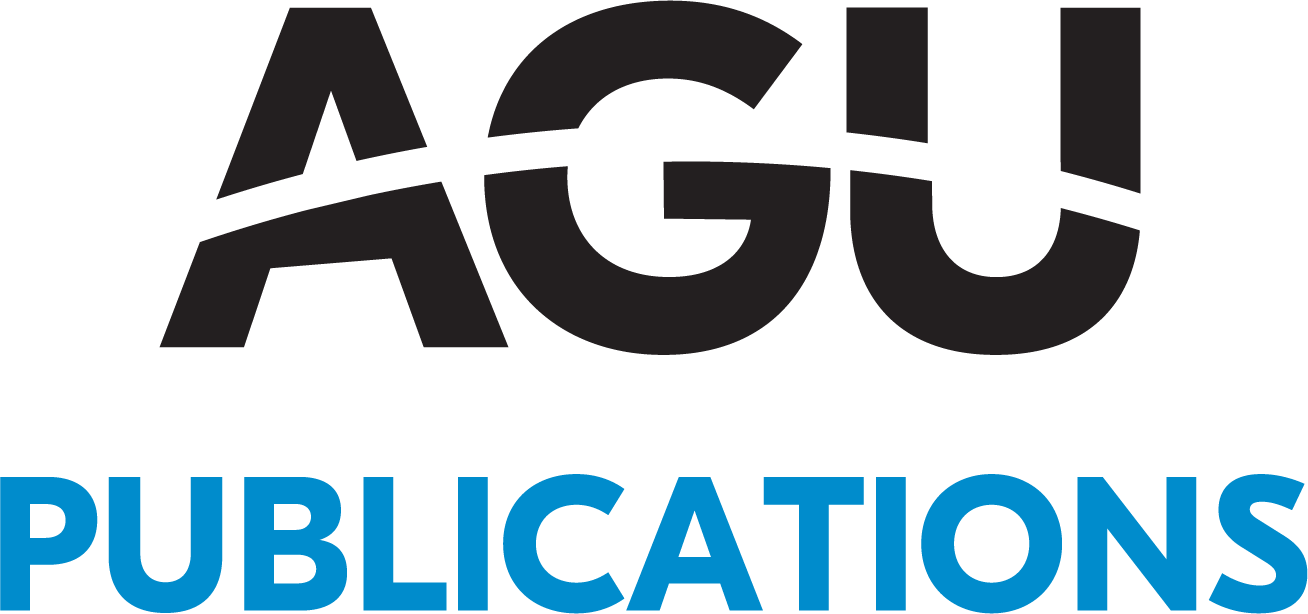


*Earth and Space Science*

Supplementary information for

**Application of Distributed Acoustic Sensing for Future Planetary Applications: Initial Results from the San Francisco Volcanic Field, a Lunar Analogue**

Nicholas Harmon^a^, Ryan Porter^b^, Catherine Rychert^a^, Nicholas Schmerr^c^, Madison M. Smith, Zhichao Shen, Wenbo Wu, Jacob Giles^c^, Naoma McCall^d^, Jingchuan Wang^c^, Linden Wike^c^, John West^e^, Austin Hoyle^c^, and Naya Deykes^b^

^a^Woods Hole Oceanographic Institution, Woods Hole, MA

^b^Northern Arizona University, Flagstaff, AZ

^c^University of Maryland, College Park, MD

^d^NASA Goddard Space Flight Center

^e^Arizona State University

# Contents of this file

Figures S1 to S3


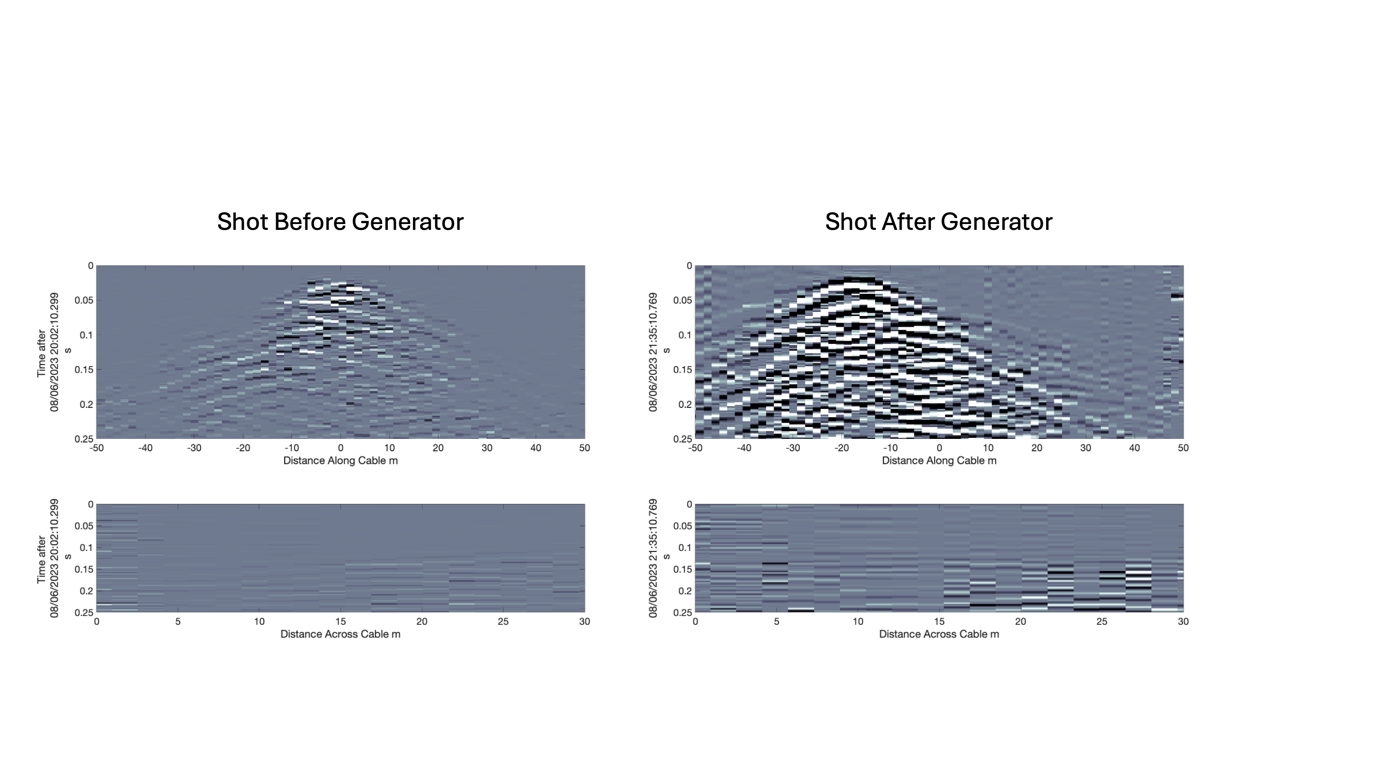


**Figure S1. Examples of shots before (left panels) and after (right panels) the generator was turned on for the Onyx Interrogator at the Double Crater Flow site. Top panels show the main line direction of the fiber (Fig. 1) and the bottom panel show the x-line direction of the fiber. Color scale between the sections is the same.**

**
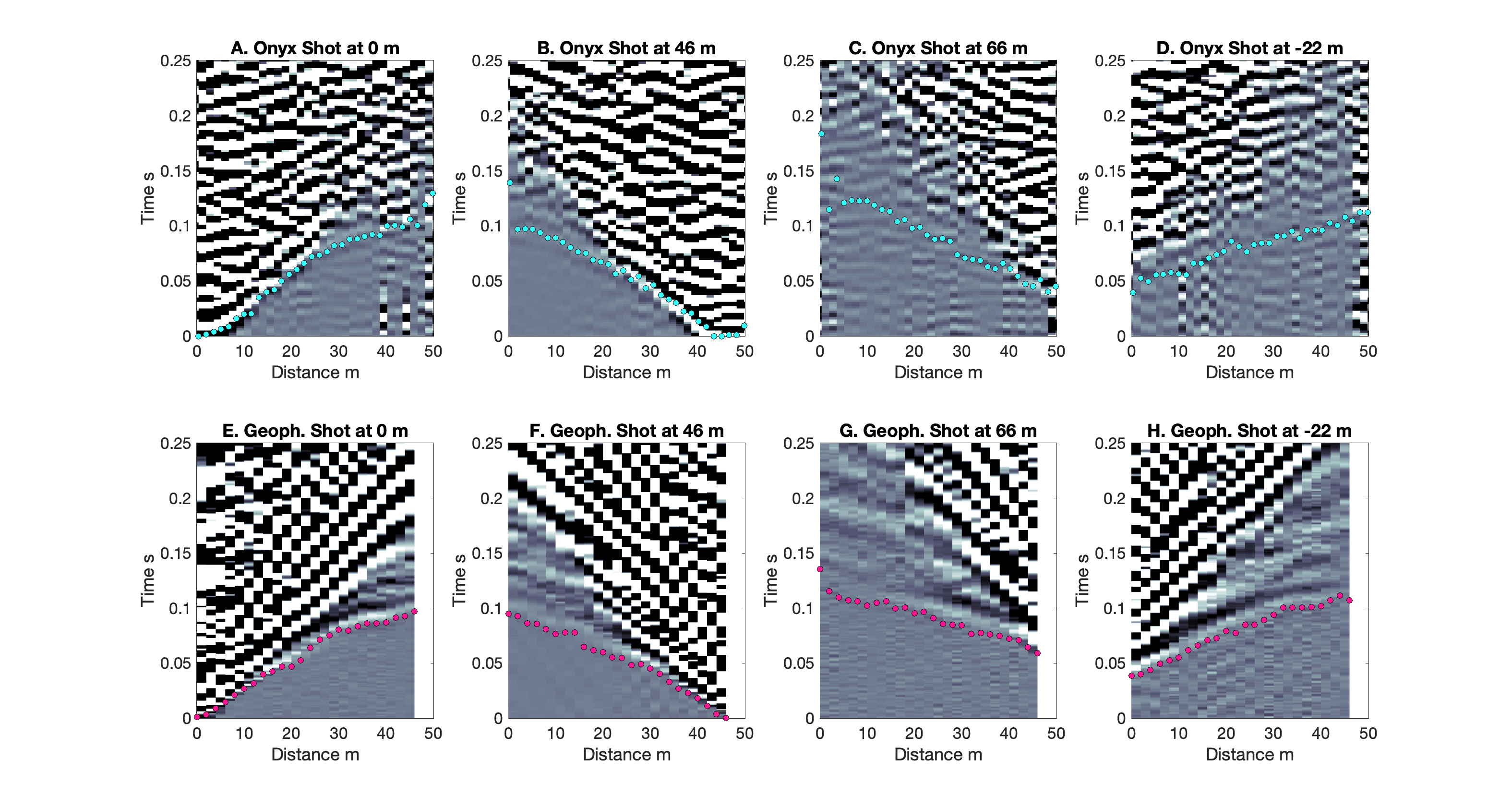
**

**Figure S2. Detail of refraction first arrival picks. Top panels (A-D) show Onyx Interrogator shot sections as the gray scale background, with first arrival picks show as cyan circles. Bottom panels (E-H) show the shot sections for the geophones, with the magenta circles showing first arrival picks. The generator was on for the shots at -22 m (D and H), which produces more coherent noise prior to the shot arrivals.**

**
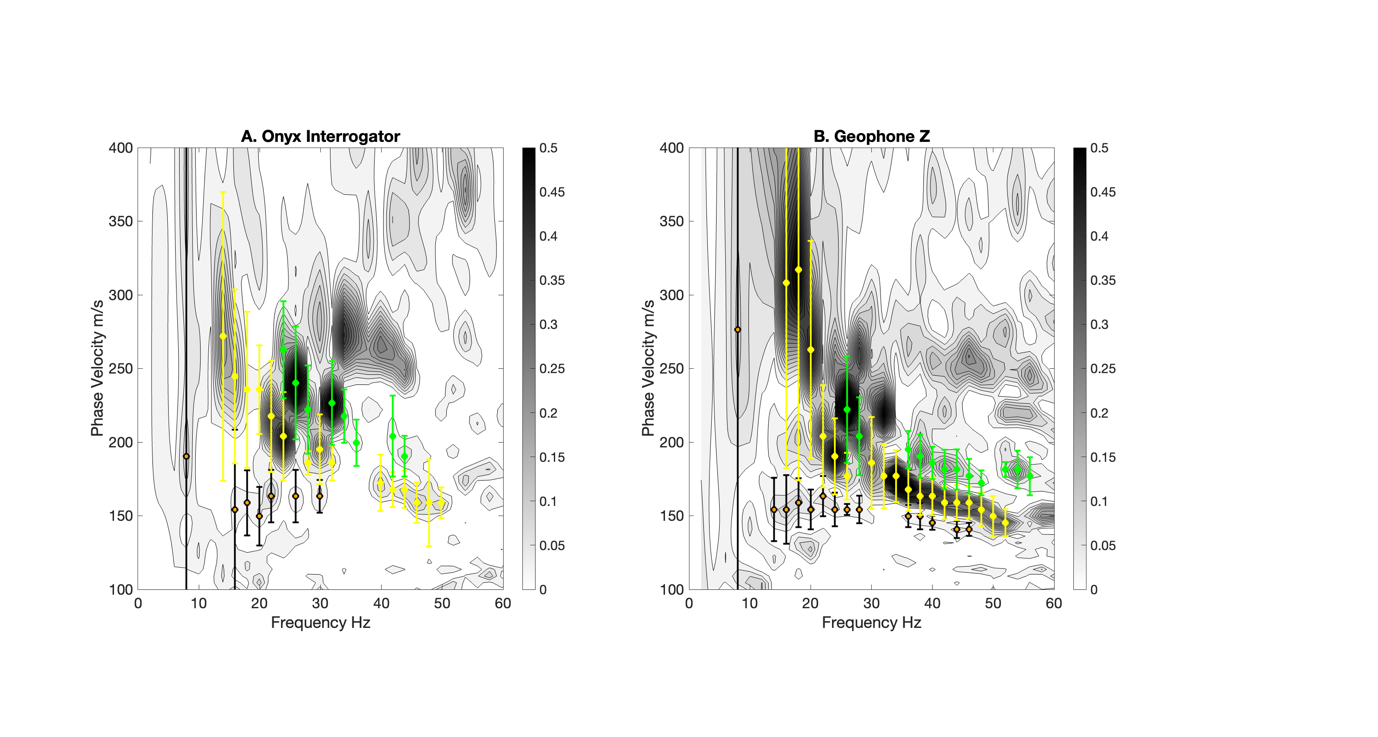
**

**Figure S3. Detail of MASW picks and errorbars. A. Onyx interrogator phase velocity vs frequency plot, fundamental mode picks are shown as orange diamonds, 1^st^ mode is shown as yellow diamonds and 2^nd^ mode is shown as green diamonds. Error bars are determined based on the width of the peak energy. B. same but for the vertical geophones.**
